# Supplementary material for: Modeling glioblastoma heterogeneity as a dynamic network of cell states
Source: Mol Syst Biol. 2021 Sep 16;17(9):e10105. doi: 10.15252/msb.202010105 (PMC8444284; doi:10.15252/msb.202010105)
Supplement: Supplementary file 6 — Source Data for Figure 5 [file MSB-17-e10105-s004.zip › Figure5A_sourcedata/GSEA_3017/nosigdb_stateF.GseaPreranked.1622795015644/RICHARDS_ZHONG_NATURE_2018_OPC_UPREG.html]

Details for gene set RICHARDS\_ZHONG\_NATURE\_2018\_OPC\_UPREG[GSEA]

|  || Dataset | state33017 |
| Phenotype | NoPhenotypeAvailable |
| Upregulated in class | na\_pos |
| GeneSet | RICHARDS\_ZHONG\_NATURE\_2018\_OPC\_UPREG |
| Enrichment Score (ES) | 0.5624727 |
| Normalized Enrichment Score (NES) | 2.9499516 |
| Nominal p-value | 0.0 |
| FDR q-value | 0.0 |
| FWER p-Value | 0.0 |
Table: GSEA Results Summary

  

Fig 1: Enrichment plot: RICHARDS\_ZHONG\_NATURE\_2018\_OPC\_UPREG      
 Profile of the Running ES Score & Positions of GeneSet Members on the Rank Ordered List

  

| PROBE | GENE SYMBOL | GENE\_TITLE | RANK IN GENE LIST | RANK METRIC SCORE | RUNNING ES | CORE ENRICHMENT || 1 | MT3 |  |  | 1 | 2.229 | 0.0759 | Yes |
| 2 | CST3 |  |  | 15 | 1.273 | 0.1136 | Yes |
| 3 | S100B |  |  | 20 | 1.167 | 0.1518 | Yes |
| 4 | RAMP1 |  |  | 24 | 1.069 | 0.1871 | Yes |
| 5 | CSPG5 |  |  | 30 | 1.044 | 0.2206 | Yes |
| 6 | EDNRB |  |  | 44 | 0.957 | 0.2475 | Yes |
| 7 | SPARC |  |  | 52 | 0.929 | 0.2762 | Yes |
| 8 | TSC22D4 |  |  | 64 | 0.855 | 0.3005 | Yes |
| 9 | PON2 |  |  | 93 | 0.764 | 0.3140 | Yes |
| 10 | LHFPL3 |  |  | 98 | 0.755 | 0.3380 | Yes |
| 11 | LUZP2 |  |  | 106 | 0.742 | 0.3603 | Yes |
| 12 | B2M |  |  | 110 | 0.736 | 0.3841 | Yes |
| 13 | GNG7 |  |  | 115 | 0.726 | 0.4072 | Yes |
| 14 | CADM2 |  |  | 137 | 0.687 | 0.4212 | Yes |
| 15 | FIBIN |  |  | 147 | 0.673 | 0.4402 | Yes |
| 16 | TSPAN7 |  |  | 148 | 0.671 | 0.4632 | Yes |
| 17 | ITM2C |  |  | 160 | 0.649 | 0.4804 | Yes |
| 18 | OMG |  |  | 252 | 0.550 | 0.4580 | Yes |
| 19 | BCAN |  |  | 302 | 0.510 | 0.4532 | Yes |
| 20 | SCD5 |  |  | 306 | 0.508 | 0.4693 | Yes |
| 21 | PTPRZ1 |  |  | 310 | 0.505 | 0.4852 | Yes |
| 22 | PSAT1 |  |  | 329 | 0.496 | 0.4941 | Yes |
| 23 | SEMA5A |  |  | 336 | 0.494 | 0.5083 | Yes |
| 24 | MMP2 |  |  | 357 | 0.485 | 0.5158 | Yes |
| 25 | GATM |  |  | 375 | 0.480 | 0.5245 | Yes |
| 26 | SPRY4 |  |  | 443 | 0.453 | 0.5097 | Yes |
| 27 | TTYH1 |  |  | 457 | 0.448 | 0.5191 | Yes |
| 28 | RFTN2 |  |  | 485 | 0.439 | 0.5219 | Yes |
| 29 | C2orf80 |  |  | 486 | 0.439 | 0.5370 | Yes |
| 30 | GPM6B |  |  | 553 | 0.413 | 0.5212 | Yes |
| 31 | PMP2 |  |  | 562 | 0.411 | 0.5316 | Yes |
| 32 | RAB31 |  |  | 580 | 0.404 | 0.5377 | Yes |
| 33 | PLEKHB1 |  |  | 597 | 0.399 | 0.5441 | Yes |
| 34 | LSAMP |  |  | 611 | 0.394 | 0.5517 | Yes |
| 35 | APOD |  |  | 636 | 0.389 | 0.5542 | Yes |
| 36 | SEZ6L |  |  | 657 | 0.383 | 0.5582 | Yes |
| 37 | BCHE |  |  | 677 | 0.376 | 0.5625 | Yes |
| 38 | ARL4A |  |  | 889 | 0.328 | 0.4780 | No |
| 39 | NKAIN4 |  |  | 923 | 0.322 | 0.4740 | No |
| 40 | SLITRK2 |  |  | 967 | 0.315 | 0.4653 | No |
| 41 | ITGB8 |  |  | 970 | 0.314 | 0.4752 | No |
| 42 | SIRT2 |  |  | 1018 | 0.307 | 0.4644 | No |
| 43 | SERPINE2 |  |  | 1030 | 0.304 | 0.4698 | No |
| 44 | SCRG1 |  |  | 1032 | 0.304 | 0.4797 | No |
| 45 | CD9 |  |  | 1070 | 0.297 | 0.4731 | No |
| 46 | PDE4B |  |  | 1404 | 0.256 | 0.3308 | No |
| 47 | KANK1 |  |  | 1449 | 0.252 | 0.3195 | No |
| 48 | PDGFRA |  |  | 1601 | -0.286 | 0.2607 | No |
| 49 | LIMA1 |  |  | 1899 | -0.421 | 0.1404 | No |
| 50 | EPB41L2 |  |  | 2067 | -0.576 | 0.0844 | No |
Table: GSEA details [plain text format]

  

Fig 2: RICHARDS\_ZHONG\_NATURE\_2018\_OPC\_UPREG: Random ES distribution      
 Gene set null distribution of ES for **RICHARDS\_ZHONG\_NATURE\_2018\_OPC\_UPREG**
